# Linear Peptide Epitopes Derived from ErpP, p35, and FlaB in the Serodiagnosis of Lyme Disease

**DOI:** 10.3390/pathogens11080944

**Published:** 2022-08-20

**Authors:** Paul M. Arnaboldi, Adiya S. Katseff, Mariya Sambir, Raymond J. Dattwyler

**Affiliations:** 1 Department of Pathology, Microbiology, and Immunology, New York Medical College, Valhalla, NY 10595, USA; 2 Biopeptides, Corp., East Setauket, NY 11733, USA

**Keywords:** Lyme disease, *Borrelia burgdorferi*, linear epitopes

## Abstract

Lyme disease is the most common vector-borne disease in the northern hemisphere. Current serodiagnostics are insensitive in early infection. Sensitivity in these seroassays is compromised by the necessity to preserve specificity in the presence of cross-reactive epitopes in *Borrelia burgdorferi* target antigens. We evaluated the efficacy of using synthetic peptides containing epitopes unique to *B. burgdorferi* as antigen targets in a Lyme disease seroassay. We performed linear B cell epitope mapping of the proteins p35 (BBH32) and ErpP to identify unique epitopes. We generated peptides containing these newly identified linear epitope sequences along with previously identified epitopes from the antigens FlaB and VlsE and evaluated their diagnostic capabilities via ELISA using large serum sets. Single-epitope peptides, while specific, demonstrated insufficient sensitivity. However, when epitopes from FlaB, ErpP, or p35 were combined in tandem with an epitope from VlsE, the sensitivity of the assay was significantly increased without compromising specificity. The identification of additional unique epitopes from other *B. burgdorferi* antigens and the further development of a combined multi-peptide-based assay for the laboratory diagnosis of Lyme disease offers a way to address the poor specificity associated with the use of whole protein antigen targets and thus significantly improve the laboratory diagnosis of Lyme disease.

## 1. Background

Lyme disease, caused by pathogenic members of the *Borrelia burgdorferi* sensu lato genospecies, is the most common vector-borne disease in the United States and Europe [1,2]. The CDC estimates that there are more than 400,000 newly diagnosed cases of Lyme disease each year in the U.S. (http://www.cdc.gov/lyme/stats/humanCases.html accessed on 27 June 2022), with over 3 million diagnostic laboratory tests ordered each year [3,4]. Treatment of early Lyme disease with appropriate oral antibiotics is highly effective at preventing the development of disseminated sequelae in most cases. However, if the disease progresses, significant nervous system or musculoskeletal involvement can occur with the risk of permanent damage to the nervous and musculoskeletal systems [1,2,5]. Early detection and treatment are critical to ensure good patient outcomes; unfortunately, current serodiagnostics lack sensitivity in early Lyme disease detection precisely at the time when diagnosis and treatment have the maximum effect on the outcome. Erythema migrans (EM) is the characteristic skin lesion of Lyme disease. In Lyme-disease-endemic regions, EM is considered diagnostic [5,6,7,8]. However, roughly 20% of patients do not develop EM [9]. Further complicating the diagnosis is that EM is variable in appearance and may not be recognized. It is also fleeting and may be gone by the time a patient seeks medical care. Considering that 20% of patients per year (approximately 60,000 based on current CDC estimations) do not develop EM, and given the difficulty in accurately identifying all those who do develop it, sensitive and specific laboratory testing becomes critical to provide optimal patient outcomes. 

Direct detection of *B. burgdorferi* using assays including culture and/or PCR has proven ineffective in the absence of EM [6,9,10,11]. The laboratory diagnosis of Lyme disease is based on indirect methods. The mainstays for laboratory diagnosis of Lyme disease are serologic assays that detect antibodies to *B. burgdorferi* antigens. Because of the lack of specificity in past and current serologic assays, a two-tier paradigm consisting of an EIA followed by an immunoblot, or more recently, another EIA is recommended by the CDC to mitigate false positivity [5,7,8,9,10,11,12,13]. The two-tier paradigm addressed the issue of specificity, but unfortunately, due to its stringency, it is insensitive at the time many patients with early LD seek initial medical care. A 2016 meta-analysis demonstrated a low sensitivity of 46.3% for early stage Lyme disease across all the studies evaluated, with sensitivity increasing to 89.7% and 99.4% in patients with early disseminated or late-stage disease, respectively [14]. Although protein-based assays can be made to be sensitive, protein antigens commonly contain cross-reactive epitopes; thus, increased sensitivity is limited by decreased specificity. Despite extensive research, no protein-based assay has overcome this specificity problem. The use of synthetic peptides containing linear epitopes unique to *B. burgdorferi* as antigen targets can improve specificity [15,16,17,18,19,20,21,22,23,24,25,26,27] by eliminating nonspecific, cross-reactive epitopes [8,11,12,15,28,29,30,31,32]. By removing cross-reactive epitopes, it is possible to improve specificity, which could lead to the development of single-tier assays that would allow for greater sensitivity. In the present study, we evaluated the diagnostic potential of synthetic peptides from three prominent *B. burgdorferi* antigens expressed during human infection, ErpP (OspE/F related protein P), p35, and FlaB. ErpP binds complement factor H and plasminogen and is thought to play a role in bacterial survival/immune evasion and dissemination [33,34,35,36]. The second prominent antigen, p35 (BBH32), is a surface lipoprotein of unknown function [37,38]. Finally, FlaB is a major constituent of the *B. burgdorferi* flagellum [39,40]. We assessed both IgM and IgG binding using a standard ELISA comparing the diagnostic potential of immunodominant epitopes from each protein both singularly and when conjugated to an immunogenic epitope-containing peptide derived from the *B. burgdorferi* protein VlsE [17].

## 2. Results

**Figure 1:**
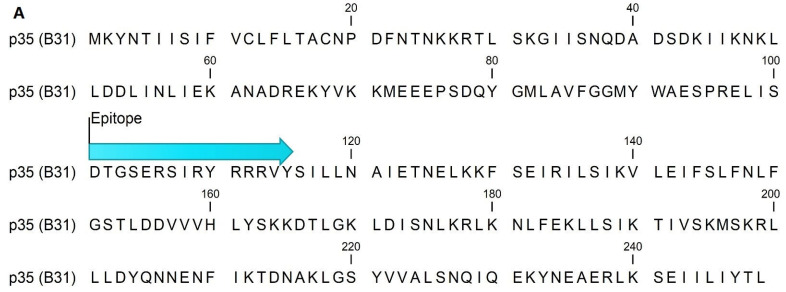

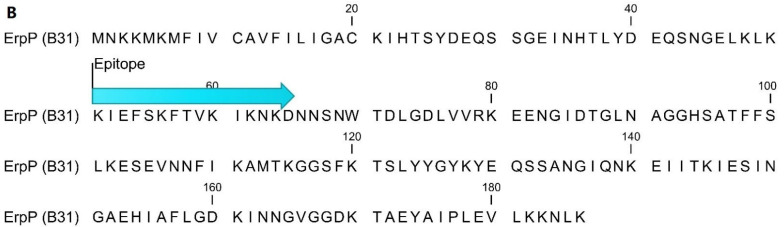
Sequence of p35 (accession WP_010256558.1) (**A**) and ErpP (accession WP_010883886.1) (**B**) from the B31 strain of *B. burgdorferi* demonstrating the location of the epitope identified by epitope mapping.

Epitope mapping of full-length ErpP and p35 revealed a single 15-AA peptide containing a linear epitope in each protein (Figure 1a,b). All eight patient sera used for epitope mapping detected p35(101–115). ErpP(51–65) was detected by four of the eight patient sera used for epitope mapping, and it was the only peptide that bound antibodies in multiple patient sera. We evaluated the efficacy of these peptides and a previously described 13AA peptide derived from Borrelia flagellin, FlaB(211–223), as antigen targets for the detection of IgM and IgG in a large panel of sera from patients with early (EM+) or late (LA+) Lyme disease in an ELISA format (Table 1). Sera from healthy individuals, patients with RA, or patients with syphilis were used as controls for nonspecificity (Table 2). Cutoffs were defined as 3SD and 2SD or positivity and equivocality, respectively, from the mean IgM or IgG binding to peptides in healthy control serum. In 13.1% and 6.3% of EM+ patient sera, respectively, p35(101–155) demonstrated positive binding of IgM and IgG. Equivocal levels of IgM and IgG were 9.0% and 13.9%, respectively. ErpP(51–65) performed marginally better, with positive binding of IgM and IgG occurring in 23.4% and 11.8% of EM+ patient sera, respectively. Equivocal binding was identical to that of p35(101–155). FlaB(211–223) detected positive levels of antibodies in a higher number of patient sera than the other two peptides; the positive detection of rate of IgM and IgG was 37.6% and 17.3%, respectively, with an equivocal detection rate of 3.0% (IgM) and 12.0% (IgG) (*p* < 0.01 FlaB(211–223) vs. p35(101–155) and ErpP(51–65) for IgM, and *p* < 0.05 FlaB(211–223) vs. p35(101–155) for IgG, FlaB(211–223) vs. ErpP(51–65), N.S for IgG). 

To enhance antibody binding detection capability, each of the three epitopes was linked to a 17-AA VlsE peptide (modVlsE(275–291)) that we previously developed. This single-epitope was modified to represent a consensus sequence to minimize the impact of sequence variability found in VlsE among different Borrelia strains [17]. In 25.9% and 54.0% of EM+ patient samples, respectively, p35(101–115)-modVlsE(275–291) (pp35-mV) demonstrated significantly higher positive binding of IgM and IgG (IgM, *p* < 0.05; IgG, *p* < 0.0001 pp35-mv vs. p35), respectively, with equivocal detection of both isotypes in 10.8% of patients. ErpP(51–65)-modVlsE(275–291) (pErpP-mV) had positive detection rates of 43.2% and 51.1% for IgM and IgG, respectively, and equivocal detection rates of 8.6% (IgM) and 10.8% (IgG) (IgM, *p* < 0.005; IgG, *p* < 0.0001 pErpP-mv vs. pErpP). FlaB(211–223)-modVlsE(275–291) (pFlaB-mV) had the highest rates of detection, positively identifying 55.5% and 54.7% of IgM and IgG in EM+ patient sera and equivocal detection rates of 5.1% (IgM) and 3.6% (IgG) (IgM, *p* < 0.005; IgG, *p* < 0.0001 pErpP-mv vs. pErpP). Late Lyme disease antibody binding detection was better with dual-epitope peptides compared to single-epitope peptides (Table 1). IgG was the predominant isotype detected, as would be anticipated for late Lyme disease samples. IgG was positively detected in 95%, 85%, and 85% of LA serum incubated with pp35-mV, pErpP-mV, and pFlaB-mV, respectively. As with the other single-epitopes, modVlsE(275–291) (mV) alone had low sensitivity for detection of antibodies during early infection, positively identifying 12.0% and 26.5% of IgM and IgG in EM+ patient sera, with equivocal binding rates of 2.4% for IgM and 3.6% for IgG.

The serum incubated with pFlaB-mV had the highest rate of total positive antibody binding (either IgM or IgG, 62.8%) when compared to both pErpP-mV (59.0%) or pp35-mV (56.9%) (Table 1). However, pErpP-mV had the lowest rate of false-negative antibody binding (either IgM or IgG, 28.8%) when compared to the other two dual-epitope peptides (pFlaB-mV, 31.4%; pp35-mV, 32.9%) due to a higher rate of equivocal binding of patient antibodies (Table 1). Using ROC analysis to compare IgM and IgG binding in EM patient sera to all negative control sera (syphilis and RA patient sera, and sera from healthy individuals with no history of Lyme disease), specificity and sensitivity at 3SD from the mean antibody binding of healthy controls were determined (Table 3). Specificities for both IgM and IgG peptide binding were greater than 95% for all peptides, except for pErpP-mV, which had a specificity of 94.9% for IgM binding. This was due to IgM binding in 5 of 38 sera from patients with RA (Table 3). In total, the use of the tandem di-epitope-containing peptides significantly increased the serodetection of anti-Borrelial antibodies compared to single peptides with a minimal effect on specificity.

## 3. Discussion

In the present study, we performed epitope mapping of two *B. burgdorferi* proteins, p35 (BBH32) and ErpP, to identify linear B cell epitopes that might serve as sensitive and specific diagnostics for early and late infection. While some antigens we have evaluated contained numerous epitopes [20,24,26,41], these proteins each contained only one linear epitope that was identified by at least 50% of the serum samples used for mapping. We also evaluated a previously identified linear epitope from the *B. burgdorferi* primary flagellin protein, FlaB. This epitope was previously shown to be unique and specific for *B. burgdorferi* (as most of the flagellin sequence is highly conserved in other bacteria) [42]. Linear epitopes were pursued in this study because they can be easily synthesized; they are not dependent on potentially complex folded structures such as conformational epitopes, and they can be more easily screened for similar sequences in other antigens that may give rise to cross-reactive antibodies and increased nonspecificity. Though somewhat counterintuitive, linear epitopes do not need to be surface-exposed to be useful diagnostically, as they may become exposed as antigens are shed and/or degraded during infection. The C6 epitope is an example of this, as it is not surface-exposed in the folded VlsE protein on the bacterial surface [21]. ErpP(51–65), p35(101–115), and FlaB(211–223) were not very sensitive for the detection of antibodies when used as single-epitope peptides. However, combining the epitopes with mVlsE(275–291), a modified version of the C6 peptide epitope [17], significantly increased antibody binding (*p* < 0.05) compared to the single-epitope peptides, with a notable increase in IgG binding. In most cases, the inclusion of the second epitope had only a modest effect on specificity compared to the individual epitopes without mVlsE(275–291) (Table 2). Single- and dual-epitope-containing peptides were screened using Lyme disease patient sera obtained from patients with physician-diagnosed Lyme disease. The pattern of antibody binding was as would be expected; the early Lyme disease sera pool (EM+) contained a mixture of IgM and IgG seropositive and seronegative patients, while the late Lyme pool contained predominantly IgG seropositive patients. This indicates that the peptides could detect disease over the course of infection. We have identified peptide epitopes in the past that were only effective in detecting early infection [19]. There is a critical need for improved diagnostics for Lyme disease. Current diagnostic assays are insensitive during early infection, the phase of the disease when treatment is most effective at preventing the dissemination of *B. burgdorferi* and the development of sequelae. Our data support that single-epitope peptides are suboptimal when used alone but that peptides can be combined to improve sensitivity while still maintaining good specificity. Though the sensitivity of the di-epitope peptides was not high enough to justify their use as single-assay targets for serodiagnosis, they could serve as one of a group of components, including as part of a multiplex-based assay [25]. Our results provide support for the identification of additional peptide antigens and the further development of combined multi-peptide-based assays for the laboratory diagnosis of Lyme disease. 

## 4. Materials and Methods

### 4.1. Patient Samples

A total of 145 early Lyme disease sera were collected at first presentation with erythema migrans from patients under informed consent with the approval of the institutional review boards of Stony Brook University in Stony Brook, NY (*n = 21*), New York Medical College in Westchester, NY (*n = 74*), and Gundersen-Lutheran Medical Center in La Crosse, WI (*n = 50*). Samples were accumulated over the course of the last 30 years. The 50 samples from Gundersen-Lutheran Medical Center in La Crosse, WI, had a clinician-documented EM lesion of > 4 cm, appropriate epidemiologic history (e.g., tick bite or exposure), and were seropositive according to a whole-cell ELISA (VIDAS by BioMerieux, Durham, NC, USA). We were not provided with the clinical laboratory results for the other deidentified Lyme disease patients beyond the fact that the patients were EM+. A total of 20 late Lyme disease samples were collected from patients at Gundersen-Lutheran Medical Center in La Crosse, WI, with Lyme arthritis (LA) (*n = 20*) that had one or more episodes of swollen joints, appropriate epidemiologic history, and positive reactivity using a whole-cell ELISA (VIDAS). The regions where the samples were collected, lower New York and Wisconsin, are highly endemic areas for Lyme disease. Sera from healthy volunteers (*n = 64*) were collected in New Mexico, which is not endemic for Lyme disease, and were purchased from Creative Testing Solutions (Tempe, AZ, USA). A total of 80 sera from patients with rheumatoid arthritis (RA, *n = 40*, rheumatoid factor status unknown) or who were Rapid Plasma Reagin positive (RPR+, first-tier test for syphilis, *n- = 35*) were purchased from Bioreclamation LLC (Westbury, NY, USA). These samples were collected in a region endemic for Lyme disease (the northeastern US). A total of 34 of the 35 RPR+ sera used in this study had positive or equivocal antibody levels against *Treponema pallidum* by ELISA (Abnova, Walnut, CA, USA). Some serum samples are not represented in all data sets because they were fully consumed during experimentation.

### 4.2. Epitope Mapping and Peptide Sequences

Overlapping peptide libraries (ProImmune, Oxford, UK), consisting of 15-AA long peptides overlapping by 10-AA, were generated using sequences of p35 (BBH32) (accession# WP_010256558.1) and ErpP (accession # WP_010883886.1) from the B31 strain of *Borrelia burgdorferi sensu stricto*. ProImmune, Inc. performed the epitope mapping under contract using their proprietary ProArray Ultra technology, as previously described [19]. Sera from 8 patients with physician-diagnosed EM+ early Lyme disease that were seropositive by two-tier testing, with an IgG immunoblot sensitivity of 9-10, of 10 bands were used for the epitope mapping. Using the 17-mer B31 sequence previously described, mod-VlsE(275–291) was engineered. [17]. It includes substitutions at AA278 (D→ N), AA282 (A→ V), and AA290 (M→ V), which represent natural sequence variability found in Borrelia strains other than B31. It is similar in sequence to the IR6 epitope of VlsE, which has been shown to be sensitive as a first- or second-tier assay target [16,27]. FlaB(211–223) is a previously described immunodominant peptide of the Borrelia Flagella [42] contained within a region of the protein previously identified to have little cross-reactivity with flagella from other species [39,40]. Peptides: p35(101–115): DTGSERSIRYRRRVY, ErpP(51–65): KIEFSKFTVKIKNKD, FlaB(211–223): (C)VQEGVQQEGAQQP, and mod-VlsE(275–291): MKKNDQIVAAIALRGVA were synthesized by Lifetein (Hillsborough, NJ, USA). Dual-epitope peptides were synthesized containing three glycine (G) residues between two epitope sequences.

### 4.3. ELISA

ELISAs were performed as previously described [19], using the following conditions: plates were coated with 10 μg/ml of peptides, sera were incubated at a 1:100 dilution, and antibody binding was detected with a 1:8000 dilution of anti-human IgM (μ-chain specific) antibodies or a 1:5000 dilution of HRP-labeled goat anti-human IgG (γ-chain specific) antibodies (Southern Biotech, Birmingham, AL, USA). Cutoffs for positive and equivocal antibody binding were defined as >3SD or >2SD but <3SD from the mean absorbance of healthy controls, respectively. IgM and IgG absorbance less than 2SD from the mean of healthy controls were considered serologically negative.

### 4.4. Statistics

Statistical analyses were performed using Prism 7.0 software (GraphPad, La Jolla, CA, USA). We analyzed categorical data with a Chi-square test. We calculated the sensitivity and specificity of each peptide by receiver operating characteristic (ROC) analysis comparing Lyme patient serum IgM and IgG binding to that in all negative controls. The cutoff value used for comparing sensitivity and specificity was 3 standard deviations (SD) above the mean of the healthy controls. We considered *p* values of less than 0.05 statistically significant.

## Figures and Tables

**Table 1:** Positive and equivocal antibody binding to single- and dual-epitope peptides in early and late Lyme disease serum.

		Early Lyme (EM+) ^1^ (*n = as Indicated*)			Late Lyme (LA) ^2^ (*n = as Indicated*)		
		IgM	IgG	IgM + IgG ^3^	IgM	IgG	IgM + IgG
**p35(101–115)**	**Positive ^4^**	19/145 13.1%	9/144 6.3%	24/145 16.6%	0/20 0.0%	3/20 15.0%	3/20 15.0%
	**Equivocal ^5^**	13/145 9.0%	20/144 13.9%	25/145 17.2%	1/20 5.0%	7/20 35.0%	7/20 35.0%
	**Negative ^6^**	113/145 77.9%	115/144 79.9%	96/145 66.2%	19/20 95.0%	10/20 50.0%	10/20 50.0%
**pp35-mV**	**Positive**	36/139 25.9%	75/139 54.0%	78/139 56.9%	2/20 10.0%	19/20 95.0%	19/20 95.0%
	**Equivocal**	15/139 10.8%	15/139 10.8%	14/139 10.2%	0/20 0.0%	1/20 5.0%	1/20 5.0%
	**Negative**	88/139 63.3%	49/139 35.3%	45/139 32.8%	18/20 90.0%	0/20 0.0%	0/20 0.0%
**ErpP(51–65)**	**Positive**	34/145 23.4%	17/144 11.8%	45/145 31.0%	3/20 15.0%	9/20 45.0%	10/20 50.0%
	**Equivocal**	13/145 9.0%	20/144 13.9%	22/145 15.2%	2/20 10.0%	2/20 10.0%	3/20 15.0%
	**Negative**	98/145 67.6%	107/144 74.3%	78/145 53.8%	15/20 75.0%	9/20 45.0%	7/20 35.0%
**pErpP-mV**	**Positive**	60/139 43.2%	71/139 51.1%	82/139 59.0%	1/20 5.0%	17/20 85.0%	17/20 85.0%
	**Equivocal**	12/139 8.6%	15/139 10.8%	17/139 12.2%	1/20 5.0%	1/20 5.0%	1/20 5.0%
	**Negative**	67/139 48.2%	53/139 38.1%	40/139 28.8%	18/20 90.0%	2/20 10.0%	2/20 10.0%
**FlaB(211–223)**	**Positive**	50/133 37.6%	23/133 17.3%	60/133 45.1%	2/20 10.0%	15/20 75.0%	15/20 75.0%
	**Equivocal**	4/133 3.0%	16/133 12.0%	10/133 7.5%	0/20 0.0%	1/20 5.0%	1/20 5.0%
	**Negative**	79/133 59.4%	94/133 70.7%	63/133 47.4%	18/20 90.0%	4/20 20.0%	4/20 20.0%
**pFlaB-mV**	**Positive**	76/137 55.5%	75/137 54.7%	86/137 62.8%	7/20 35.0%	17/20 85.0%	17/20 85.0%
	**Equivocal**	7/137 5.1%	5/137 3.6%	9/137 6.6%	3/20 15.0%	2/20 10.0%	2/20 10.0%
	**Negative**	54/137 39.4%	57/137 41.6%	42/137 30.7%	10/20 50.0%	1/20 5.0%	1/20 5.0%
**mVlsE(275–291) (mV)**	**Positive**	10/83 12.0%	22/83 26.5%	23/83 27.7%	1/19 5.3%	13/19 68.4%	13/19 68.4%
	**Equivocal**	2/83 2.4%	3/83 3.6%	3/83 3.6%	0/19 0.0%	1/19 5.3%	1/19 5.3%
	**Negative**	71/83 85.6%	58/83 69.9%	57/83 68.7%	18/19 94.7%	5/19 26.3%	5/19 26.3%

^1^-Sera from patients obtained during the first visit with EM; ^2^-sera from patients with one or more episodes of swollen joints, appropriate epidemiologic history, and positive reactivity by whole-cell ELISA (VIDAS); ^3^-positive for either IgM, IgG, or both; ^4^-greater than 3SD from the mean of healthy control sera; ^5^-greater than 2SD but less than 3SD from the mean of healthy control sera; ^6^-less than 2SD from the mean of healthy control sera.

**Table 2:** Positive and equivocal antibody binding to single- and dual-epitope peptides in healthy control, RA, and syphilis patient serum.

		Healthy Controls ^1^			RA ^2^			Syphilis ^3^		
		IgM	IgG	IgM + IgG ^4^	IgM	IgG	IgM + IgG	IgM	IgG	IgM + IgG
**p35(101–115)**	**Positive ^5^**	0/64 0.0%	0/64 0.0%	0/64 0.0%	0/40 0.0%	2/40 5.0%	2/40 5.0%	1/35 2.9%	3/35 8.6%	4/35 11.4%
	**Equivocal ^6^**	2/64 3.1%	2/64 3.1%	4/64 6.3%	0/40 0.0%	3/40 7.5%	3/40 7.5%	1/35 2.9%	6/35 17.1%	7/35 20.0%
	**Negative ^7^**	62/64 96.9%	62/64 96.9%	60/64 93.8%	40/40 100.0%	35/40 87.5%	35/40 87.5%	33/35 94.3%	26/35 74.3%	24/35 68.6%
**pp35-mV**	**Positive**	0/64 0.0%	0/64 0.0%	0/64 0.0%	1/38 2.6%	2/38 5.3%	3/38 7.9%	0/34 0.0%	4/34 11.8%	4/34 11.8%
	**Equivocal**	3/64 4.7%	4/64 6.3%	6/64 9.4%	2/38 5.3%	6/38 15.8%	8/38 21.1%	0/34 0.0%	8/34 23.5%	8/34 23.5%
	**Negative**	61/64 95.3%	60/64 93.8%	58/64 90.6%	35/38 92.1%	30/38 78.9%	27/38 71.1%	34/34 100.0%	22/34 64.7%	22/34 64.7%
**ErpP(51–65)**	**Positive**	0/64 0.0%	0/64 0.0%	0/64 0.0%	0/40 0.0%	0/40 0.0%	0/40 0.0%	2/35 5.7%	1/35 2.9%	3/35 8.6%
	**Equivocal**	3/64 4.7%	1/64 1.6%	4/64 6.3%	4/40 10.0%	1/40 2.6%	5/40 12.8%	0/35 0.0%	3/35 8.6%	3/35 8.6%
	**Negative**	61/64 95.3%	63/64 98.4%	60/64 93.8%	36/40 90.0%	38/40 97.4%	34/40 87.2%	33/35 94.3%	31/35 88.6%	29/35 82.9%
**pErpP-mV**	**Positive**	1/64 1.6%	1/64 1.6%	1/64 1.6%	5/38 13.2%	0/38 0.0%	5/38 13.2%	1/34 2.9%	1/34 2.9%	2/34 5.9%
	**Equivocal**	4/64 6.3%	2/64 3.1%	6/64 9.4%	2/38 5.3%	2/38 5.3%	4/38 10.5%	3/34 8.8%	0/34 0.0%	3/34 8.8%
	**Negative**	59/64 92.2%	61/64 95.3%	57/64 89.1%	31/38 81.6%	36/38 94.7%	29/38 76.3%	30/34 88.2%	33/34 97.1%	29/34 85.3%
**FlaB(211–223)**	**Positive**	1/64 1.6%	0/64 0.0%	1/64 1.6%	0/44 0.0%	2/44 4.5%	2/44 4.5%	0/34 0.0%	2/34 5.9%	2/34 5.9%
	**Equivocal**	2/64 3.1%	2/64 3.1%	3/64 4.7%	1/44 2.3%	1/44 2.3%	2/44 4.5%	1/34 2.9%	3/34 8.8%	4/34 11.8%
	**Negative**	61/64 95.3%	62/64 96.9%	60/64 93.8%	43/44 97.7%	41/44 93.2%	40/44 90.9%	33/34 97.1%	29/34 85.3%	28/34 82.4%
**pFlaB-mV**	**Positive**	2/64 3.1%	2/64 3.1%	4/64 6.3%	2/40 4.2%	1/40 2.1%	3/40 6.3%	1/32 3.1%	1/32 3.1%	2/32 6.3%
	**Equivocal**	1/64 1.6%	2/64 3.1%	3/64 4.7%	5/40 10.4%	1/40 2.1%	6/40 12.5%	1/32 3.1%	1/32 3.1%	2/32 6.3%
	**Negative**	61/64 95.3%	60/64 93.7%	57/64 89.1%	41/40 85.4%	46/40 95.8%	39/40 81.3%	30/32 93.7%	30/32 93.7%	28/32 87.5%
**mVlsE(275–291) (mV)**	**Positive**	0/61 0.0%	1/61 1.6%	1/61 1.6%	0/30 0.0%	0/30 0.0%	0/30 0.0%	0/20 0.0%	4/20 20.0%	4/20 20.0%
	**Equivocal**	4/61 6.6%	2/61 3.3%	5/61 8.2%	0/30 0.0%	0/30 0.0%	0/30 0.0%	0/20 0.0%	3/20 15.0%	3/20 15.0%
	**Negative**	57/61 93.4%	58/61 95.1%	55/61 90.2%	30/30 100.0%	30/30 100.0%	30/30 100.0%	20/20 100.0%	13/20 65.0%	13/20 65.0%

^1^-Sera from healthy individuals living in an area not endemic for Lyme disease (New Mexico); ^2^-sera from patients with rheumatoid arthritis (RF factor unknown); ^3^-sera from patients with syphilis; ^4^-positive for either IgM, IgG, or both; ^5^-greater than 3SD from the mean of healthy control sera; ^6^-greater than 2SD but less than 3SD from the mean of healthy control sera; ^7^-less than 2SD from the mean of healthy control sera.

**Table 3:** Sensitivity and specificity of single- and dual-epitope peptides in ELISA ^1^.

	IgM		IgG	
	Sensitivity	Specificity	Sensitivity	Specificity
**p35(101–115)**	13.8%	99.3%	6.9%	96.4%
**pp35-mV**	35.3%	99.3%	54.0%	95.6%
**ErpP(51–65)**	24.1%	98.6%	12.5%	99.3%
**pErpP-mV**	43.2%	94.9%	51.8%	98.5%
**FlaB(211–223)**	37.6%	98.6%	17.3%	97.2%
**pFlaB-mV**	56.2%	96.5%	54.7%	97.9%
**mVlsE(275–291) (mV)**	12.1%	99.1%	26.5%	95.5%

^1^-Specificity and sensitivity were determined using ROC analysis comparing antibody binding in early Lyme patient sera to negative control sera (healthy control, RA, and syphilis) at >3SD from the mean of healthy control sera.

## Data Availability

Not applicable.
